# Pronking and bounding allow a fast escape across a grassland populated by scattered obstacles

**DOI:** 10.1098/rsos.230587

**Published:** 2023-09-13

**Authors:** Francesco Righini, Marina Carpineti, Fabio Giavazzi, Alberto Vailati

**Affiliations:** ^1^ Dipartimento di Fisica A. Pontremoli, Università degli Studi di Milano, 20133 Milano, Italy; ^2^ Dipartimento di Biotecnologie Mediche e Medicina Traslazionale, Università degli Studi di Milano, 20133 Milano, Italy

**Keywords:** animal movement, stotting, pronking, ‌bounding, predation and escape‌, ballistics, leap angle, robotic exploration

## Abstract

Some quadrupeds have evolved the ability of pronking, which consists in leaping by extending the four limbs simultaneously. Pronking is typically observed in some ungulate species inhabiting grassland populated by obstacles such as shrubs, rocks and fallen branches scattered across the environment. Several possible explanations have been proposed for this peculiar behaviour, including the honest signalling of the fitness of the individual to predators or the transmission of a warning alert to conspecifics, but so far none of them has been advocated as conclusive. In this work, we investigate the kinematics of pronking on a two-dimensional landscape populated by randomly scattered obstacles. We show that when the density of obstacles is larger than a critical threshold, pronking becomes the gait that maximizes the probability of trespassing in the shortest possible time all the obstacles distributed across the distance fled, and thus represents an effective escape strategy based on a simple open-loop control. The transition between pronking and more conventional gaits such as trotting and galloping occurs at a threshold obstacle density and is continuous for a non-increasing monotone distribution of the height of obstacles, and discrete when the distribution is peaked at a non-zero height. We discuss the implications of our results for the autonomous robotic exploration on unstructured terrain.

## 1. Introduction

Animals have evolved different modes of legged locomotion in the environment surrounding them [1], with a strong specialization of the locomotor system dictated by the needs of predation and escape [2]. On land, galloping represents an effective strategy to achieve a fast horizontal speed, which on a flat landscape maximizes the probability of a prey surviving a hunt, and for a predator the probability of a successful catch. In general, galloping allows the stride to be directed as much as possible horizontally and to maximize stride length, but a purely horizontal thrust is prevented by the bio-mechanical structure of the animal, by need of supporting its weight, and by the need of providing a reaction of the terrain [3]. When the environment is populated by obstacles of size comparable to or larger than that of the animal, leaping becomes an effective moving strategy that allows it to achieve a fast displacement, while simultaneously avoiding being stuck by obstacles. Pronking, or stotting, is a peculiar behaviour adopted by some mammalians that consists of repeatedly extending the four limbs simultaneously to achieve a sequence of leaps. Pronking has evolved independently in several animal species across the globe, including artiodactyls like the Columbian black-tailed deer and the mule deer in northern America [4,5], the Thomson gazelle in Africa [6] and rodents like mara in South America [7]. Pronking has also been reported in eight legged opilionids like harvestmen, which adopt it as an escape manoeuver [8]. Some other species, including jumping mouse, woodland mouse, wild hare and many others, adopt an escape strategy based on a bounding gait, which consists of the simultaneous extension of the hind legs that is used to leap repeatedly. The high energy loss determined by the impact with the terrain does not make pronking and bounding efficient gaits to travel rapidly in an obstacle ridden environment [9]. Indeed, the motivation for pronking is still debated in the scientific community [10], and several possible explanations have been proposed [11], among which are the signalling to con-specifics of the presence of predators [12], an honest signal to predators of the fitness of the potential prey [13] and a fast escape from predators on a rugged terrain [14]. A common feature of the species that exhibit pronking and bounding is that they typically inhabit open environments, such as flat grassland populated by sparse natural obstacles, and that they exhibit cursorial adaptations aimed at predation avoidance. In this work, we develop a minimal kinematic model for the escape of an animal based on the selection between two possible locomotor gaits. The two gaits consist either of a sequence of forward steps directed horizontally to maximize speed, as occurs in the case of running, trotting and galloping, or in a sequence of angled leaps that allow the animal to overpass obstacles, as in the case of pronking and bounding. We consider the limit case of an arbitrarily small duty cycle of the stride, where a nearly instantaneous propulsive phase is followed by a ballistic phase that dominates the duration and length of the stride. Under this assumption, the motion of the animal can be mostly entirely described kinematically as a sequence of ballistic phases. We assume that the modulus of the velocity *v*_0_ at the end of the propulsive phase of each step or leap is fixed, while the take-off angle *θ* formed with the horizontal plane can change. We consider the real case of mammalians of medium–large size with a large Reynolds number and a small Froude number, a condition that allows neglecting the drag exerted by air during the jump [15,16]. A fast horizontal speed of locomotion and the ability of overpassing a series of obstacles are the two key factors that determine the survival of a prey during an escape run. However, the maximization of one of these two parameters is in conflict with the maximization of the other. Indeed, the successful fleeing of a prey from a predator requires jumping over obstacles to avoid being slowed down or stuck by them. In general, visual clues can largely help negotiate an obstacle, with the drawback that the presence of multiple obstacles requires a very fast processing of these clues from the nervous system of the animal. Conversely, an open-loop escape strategy like the one discussed in our work relies on the fast automatic replication of a set of actions, in our case a set of identical leaps, which maximizes statistically the probability of survival, without the need for an active processing of information on the geometry of the environment. A meaningful example of an open-loop escape strategy is represented by the free fall of the pebble toad. When chased by a predator, the toad folds its limbs to assume a compact rounded shape, and lets its body fall down the cliffs where it lives. This manoeuver determines a fast displacement of the animal that does not require a prior knowledge of the geometric details of the environment. This manoeuver is not routinely adopted by the toad to move over the terrain, but only in the case of danger, due to the high stress imposed by it on the body of the animal. More generally, when escaping animals can adopt highly costly strategies, including caudal autotomy, which largely affects the subsequent deambulation capabilities of the animal. For these reasons, in our analysis we do not take into consideration a cost for the adoption of an escape strategy.

When fleeing with a sequence of leaps, the best open-loop strategy to overcome large obstacles is to jump with a take-off angle close to 90° with respect to the horizontal plane, because this is the condition that maximizes the height reached during the aerial phase of the jump. This escape strategy has the major drawback that the thrust is directed vertically and does not allow a horizontal displacement of the prey. Conversely, the horizontal speed is maximized in the presence of a purely horizontal thrust for a take-off angle of 0°, a strategy that does not allow any obstacle to be overcome.

We show that a suitable figure of merit for the survival of preys during an escape run on a landscape populated by random obstacles is represented by the product of the horizontal component of the velocity and of the probability of overpassing a sequence of obstacles. The maximization of this figure of merit yields the result that when the density of obstacles is larger than a critical threshold pronking becomes the most effective escape strategy, while for densities smaller than the threshold gaits like galloping that optimize horizontal thrust are more effective. We discuss how the features of the probability distribution for the height of obstacles affect the nature of the transition. In the presence of a non-increasing monotone distribution, the transition is continuous, while in the presence of a single-peaked distribution with maximum value at a finite non-zero height of the obstacle the transition is discrete.

The investigation of animal gaits has bio-mimetic applications to the development of robots with versatile motion, able to adapt to different needs [17–23]. Pronking is one of the most-studied gaits, as it allows for obtaining a fast and stable motion with a limited number of control parameters, due to the fact that all the legs are parallel and move in phase [18,24–27]. In the case of movement in an uneven environment [28–32], pronking can be a particularly effective gait to manage the problem of over-passing obstacles [19,31]. The results of our work could pave the way for further applications of pronking as a favourite choice for the development of robots that have to move in a terrain with randomly distributed natural boundaries.

## 2. Model

### 2.1. Kinematics

We consider a population of animals inhabiting a flat landscape populated by random obstacles. When a specimen is chased by a predator, it escapes by moving in a prescribed direction through a sequence of identical leaps. Each leap is preceded by a short thrust phase that brings the animal to an initial velocity **v_0_** characterized by a take-off angle *θ* with respect to the horizontal ground plane. Neglecting air drag, the equations of motion for each leap are

2.1 $$x(t)=x_{0}+v_{0}\;t\;\cos(\theta )$$

and

2.2 $$y(t)=v_{0}\;t\;\sin(\theta )-\frac{1}{2}\;g\;t^{2},$$

where *x* and *y* are the horizontal and vertical coordinates, *x*_0_ is the initial position of the animal on the ground at the beginning of the leap, and *g* is the acceleration of gravity. The range of each leap is *x*_*R*_ = 2 *x*_*M*_ sin (*θ*) cos (*θ*), where $x_{M}={v_{0}}^{2}/g$ is the maximum range, achieved for a take-off angle of 45${\;}^{\circ }$. The top height reached during a leap is *y*_*T*_ = *x*_*M*_ sin^2^(*θ*)/2, which achieves the maximum value *y*_*M*_ = *x*_*M*_/2 for a take-off angle *θ* = 90°. In the following, we will use *y*_*M*_ as a typical length scale of the problem.

### 2.2. Single leap in the presence of a single obstacle

**Figure 1.**
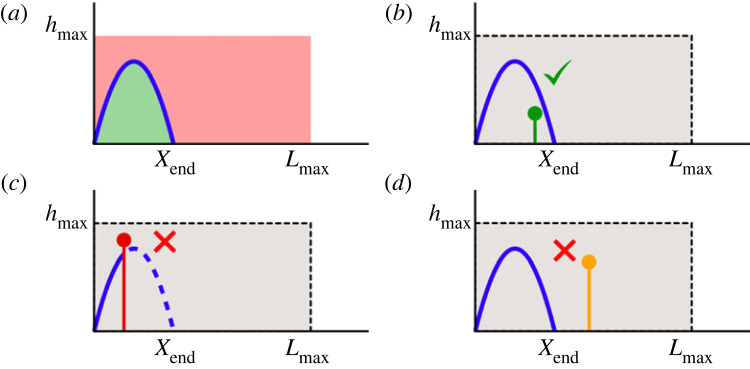
Single leap against one obstacle. The solid blue line represents a generic trajectory. (*a*) Possible position for the top edge of the obstacle during a successful jump (green region). (*b*) Successful trespass of an obstacle. (*c*) Failure due to a crash on an obstacle. (*d*) Failure due to an obstacle not reached.

We first consider the case where a single obstacle is present. The obstacle is represented by a thin vertical barrier extending from the ground up to a height *h*. We consider a leap successful if it overcomes the obstacle or, equivalently, if the top edge of the obstacle remains under the trajectory (figure 1).

The statistical properties of the obstacles are described in terms of two probability density functions *λ*(*x*) and *ρ*(*h*) governing the position of the obstacle and its height, respectively. The success probability of a leap characterized by a trajectory *y*(*x*) is thus given by

2.3 $$P=\int_{0}^{x_{R}}\left(\int_{0}^{y(x)}\rho (h)\;dh\right)\lambda (x)\;dx.$$

We assume that the probability density function *λ*(*x*) for the position of the obstacle is uniform over an interval of width *L*_max_ ≥ *x*_*M*_. Different possibilities can be considered for the height distribution of the obstacle. In the simple case where *ρ* is uniform from 0 to *h*_max_, with *h*_max_ ≥ *y*_*M*_, the success probability *P* can be calculated analytically and shown to be

2.4 $$P=\frac{8}{3}\frac{y_{M}^{2}}{h_{\max}L_{\text{max}}}\sin^{3}(\theta )\cos(\theta ).$$

In this case, *P* is simply proportional to the area below the trajectory, which is maximized by the take-off angle *θ* = 60°.

Incidentally, a leap angle of $60^{\circ }$ represents a good trade-off between the need of reaching simultaneously a jump height and range as large as possible during an escape. Larvae of species like fruit flies and gall midge and insects like froghoppers have evolved to leap with a take-off angle of $60^{\circ }$ and are able to escape by trespassing natural obstacles of sizes much larger than that of their bodies [16].

### 2.3. Single leap in the presence of multiple obstacles

When a landscape with more than one obstacle is considered, the probability of success for a single leap estimated in the previous subsection must be suitably modified. We assume that the obstacles are distributed uniformly and independently, with a certain linear number density *ν*. To be considered successful, a single leap must overcome all the obstacles present within the leap range *x*_*R*_. The number of obstacles within an interval of width *x*_*R*_ is distributed according to a Poisson distribution

2.5 $$p_{k}=\frac{\gamma^{k}}{k!}\;e^{-\gamma },$$

where *γ* = *νx*_*R*_ is the average number of obstacles encountered during a leap. If there is exactly one obstacle within the leap range, the probability *P*_*s*,1_ of overcoming it is given by equation (2.3), with *λ* = 1/*x*_*R*_, namely

2.6 $$P_{s,1}=\frac{1}{x_{\text{R}}}\int_{0}^{x_{\text{R}}}\left(\int_{0}^{y(x)}\rho (h)\;dh\right)\;dx.$$

If there are *k* independent obstacles within the leap range, the probability *P*_*s*,*k*_ to jump over all of them is thus

2.7 $$P_{s,k}=P_{s,1}^{k}.$$

Therefore, the overall probability to overcome all the obstacles encountered in a single jump is

2.8 $$\begin{array}{rl} P_{s} & =\sum_{k=0}^{+\infty }p_{k}\;P_{s,k}\\ & =\exp[\gamma (P_{s,1}-1)]. \end{array}$$

### 2.4. Multiple leaps in the presence of multiple obstacles

We finally consider the case of an escape run where a certain escape length *L* has to be covered with multiple leaps, while avoiding all the obstacles present along the way. We assume that the animal adopts an open-loop strategy not requiring any sensory feedback, where every leap has the same take-off angle *θ* and starts at the endpoint of the previous one. These subsequent hardwired identical leaps are repeated until the distance *L* is covered.

**Figure 2.**
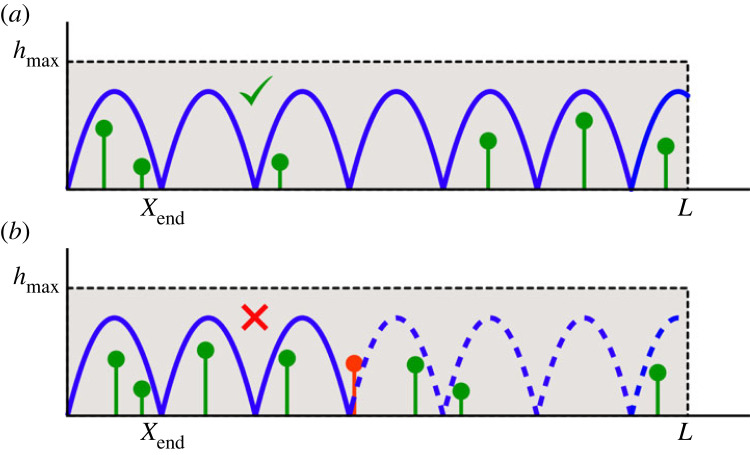
Multiple leaps in presence of many obstacles. The blue line represents a generic trajectory. (*a*) Successful case where all the obstacles are overpassed. (*b*) Failed case where a crash has occurred against the fifth obstacle.

In the presence of multiple obstacles and multiple leaps, a new definition of success should be adopted. To guarantee the survival of the animal, it is natural to define as successful the case where no obstacle is hit during the entire path (figure 2).

The whole escape path consists of a sequence of $m=\left\lceil L/x_{R}\right\rceil$ identical leaps. For the sake of simplicity, in the following we will treat *L*/*x*_*R*_ as an integer, the error introduced by this approximation becoming negligibly small in the limit *L* ≫ *x*_*M*_.

Since the obstacles are distributed uniformly and independently along the path, the success of different leaps is independent. The global success probability *P*_*e*_ of the escape can be then calculated as the success probability for a single leap given by equation (2.8) raised to the number of leaps *m*:

2.9 $$\begin{array}{rl} P_{e} & =P_{s}^{m}\\ & ={\left[\exp[\gamma (P_{s,1}-1)]\right]}^{m}\\ & =\exp[n(P_{s,1}-1)]. \end{array}$$

In the last equality, we have introduced the mean number of obstacles *n* = *γm* = *νL* encountered during the escape.

## 3. Pronking and bounding

The survival of an animal during a successful escape run requires both overpassing all the obstacles encountered along the path and moving at the maximum possible speed to avoid being reached by a predator. Jumping with a take-off angle of $90^{\circ }$ minimizes the probability of hitting an obstacle, but this is achieved at the expense of the fact that the animal does not move horizontally, Although this strategy allows the animal to maximize the number of overpassed obstacles, it leads to the immediate capture of the prey. Conversely, a take-off angle of $0^{\circ }$ allows it to move horizontally at the maximum horizontal speed *v*_*x*_ = *v*_0_, with the disadvantage that all the obstacles are hit during the run. Therefore, both these two survival strategies are completely ineffective, because they maximize only one of the two key requirements for a successful escape run, while simultaneously minimizing the other. Following these considerations, a suitable figure of merit (*FoM*) to maximize the probability of survival during an escape run is represented by the product between the global success probability *P*_*e*_ and the horizontal component of the velocity *v*_*x*_ = *v*_0_ cos (*θ*):

3.1 $$FoM\equiv v_{x}\;P_{e}.$$

In the following, we will investigate how the optimal leap angle maximizing the above-defined *FoM* depends on the density of the obstacles and on the probability distribution of their height.

### 3.1. Uniform distribution of the height of obstacles

We first consider the simple case where the probability density function of the height of obstacles is uniform in the range [0, *h*_max_]. In the following calculations, we consider *h*_max_ > *y*_*M*_.

The probability *P*_*s*,1_ of overcoming a single obstacle in a single leap has a simple analytic form

3.2 $$P_{s,1}=\frac{2}{3}\frac{y_{M}}{h_{\max}}\sin^{2}(\theta ),$$

and the *FoM* reads

3.3 $$FoM=v_{0}\cos(\theta )\;\exp\left[n\left(\frac{2}{3}\frac{y_{M}}{h_{\max}}\sin^{2}(\theta )-1\right)\right].$$

The *FoM* has an absolute maximum at the take-off angle

3.4 $$\theta =\arccos\left(\sqrt{\frac{3}{4}\frac{h_{\max}}{ny_{M}}}\right),$$

when the average number of obstacles encountered during a flee is larger than a critical threshold

3.5 $$n\geq n_{c}\;\mathrm{with}\;n_{c}=\frac{3}{4}\frac{h_{\max}}{y_{M}}$$

while *θ* = 0 for *n* < *n*_*c*_.

**Figure 3.**
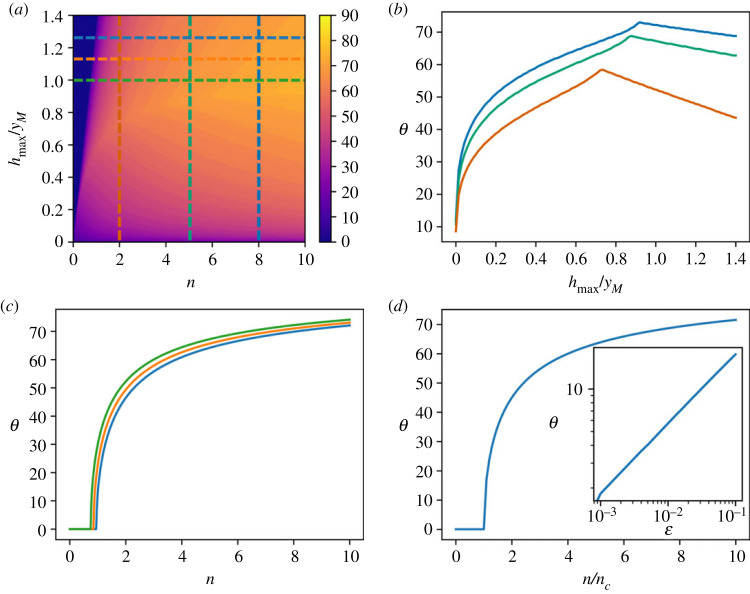
Optimal escape strategy for a uniform distribution of the obstacle height. (*a*) Optimal leap angle as a function of the average number *n* of encountered obstacles and the normalized maximum height of the obstacles *h*_max_/*y*_*M*_. (*b*) Angle that maximizes the *FoM* plotted as a function of the normalized obstacle height *h*_max_/*y*_*M*_ for different values of the average number *n* of encountered obstacles (vertical dashed lines in panel (*a*)). (*c*) Angle that maximizes the *FoM* plotted as a function of the average number *n* of encountered obstacles for different values of the normalized obstacle height *h*_max_/*y*_*M*_ (horizontal dashed lines in panel (*a*)) (*d*) Same as (*c*), where the curves are plotted as a function of the scaled variable *n*/*n*_*c*_, where *n*_*c*_ is the critical number of obstacles given in equation (3.5). Inset: power-law behaviour of the optimal leap angle as a function of the reduced number of obstacles ϵ.

Equations (3.5) and (3.4) show that the optimal leap angle is a function of the average number of obstacles *n* and the normalized maximum height of obstacles *h*_max_/*y*_*M*_. When one of these two parameters is close to zero, the best strategy for the escape is to adopt a leap angle of zero degrees, which allows the animal to trespass all the obstacles (figure 3*a*). Fixing the average number of obstacles *n* and investigating the dependence of *θ* from *h*_max_/*y*_*M*_ shows that as soon as the maximum height is slightly increased, the optimal leap angle exhibits a sharp increase, due to the divergence of its derivative in *h*_max_/*y*_M_ = 0 (figure 3*b*). For each value of *n*, there is a value for the normalized maximum height $\frac{h_{\max}}{y_{\text{M}}}$ smaller than 1 at which *θ* reaches a maximum: for higher values of *h*_max_/*y*_*M*_, obstacles can be so high that increasing the angle in order to try to overpass them is not convenient.

Keeping the maximum height of obstacles fixed, a continuous transition in the optimal take-off angle *θ* occurs when the average number of obstacles crosses the threshold value *n*_*c*_ (figure 3*c*). If *n* < *n*_*c*_, the optimal take-off velocity is directed horizontally, corresponding to running, trotting or galloping, while when the average number of obstacles exceeds the threshold, a strategy based on a sequence of jumps with a non-zero take-off angle, akin to pronking or bounding, becomes preferable.

We note that *n*_*c*_ assumes its minimum value *n*_*c*_ = 3/4 when the maximum height *h*_max_ of an obstacle matches the maximum height *y*_*M*_ reached in a vertical leap. Considering larger values of *h*_max_ shifts the threshold toward an increasingly larger average number of obstacles.

Above the threshold, the optimal take-off angle displays a sharp increase as a function of *n*, corresponding to a power law with an exponent 1/2. This can be appreciated by introducing the reduced variable $\epsilon \equiv (n-n_{c})/n_{c}$, in terms of which the optimal take-off angle exhibits a power-law behaviour with exponent 1/2 (figure 3*d*).

3.6 $$\theta =\arccos{\left(1+\epsilon \right)}^{-1/2}∼\epsilon^{1/2}.$$

### 3.2. Exponential distribution of the height of obstacles

We now consider the case where the probability density function for the heights of the obstacles is exponential and characterized by the obstacle height h* at 1/*e*:

3.7 $$\rho (h)=\frac{1}{h^{*}}e^{-(h/h^{*})}.$$

**Figure 4.**
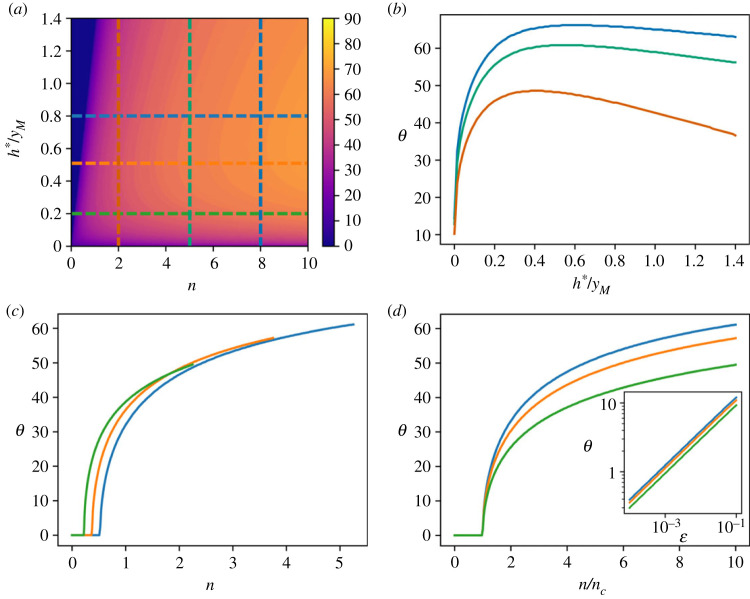
Optimal escape strategy for an exponential distribution of the obstacle height. (*a*) Optimal leap angle as a function of the average number *n* of encountered obstacles and the normalized characteristic height of the obstacles h*/*y*_*M*_. (*b*) Angle that maximizes the *FoM* plotted as a function of the normalized obstacle height h*/*y*_*M*_ for different values of the average number *n* of encountered obstacles. (*c*) Angle that maximizes the *FoM* plotted as a function of the average number *n* of encountered obstacles for different values of the normalized obstacle height h*/*y*_*M*_. (*d*) Same as (*c*), where the curves are plotted as a function of the scaled variable *n*/*n*_*c*_, where *n*_*c*_ is the critical number of obstacles given in equation (3.8). Inset: log–log plot.

In this case, the expression of *P*_*s*,1_ is quite intricate, and a closed-form expression for the optimal leap angle cannot be easily calculated. Nevertheless, we determined the optimal take-off angle by numerical maximization of the *FoM*. We found that, in close analogy with the case considered in the previous section, the optimal take-off angle exhibits a sharp transition as a function of the average number of obstacles *n* (figure 4*a*). For fixed values of *n*, as encountered in the previous case, the angle *θ* has a maximum value corresponding to a value of h*/*y*_*M*_ smaller than 1 for each *n*. Differently from the previous case, the angle *θ* as a function of h*/*y*_*M*_ is differentiable everywhere but in zero. Quite strikingly, a transition is found to occur in correspondence to the threshold value (figure 4*c*)

3.8 $$n_{c}=\frac{3}{4}\frac{h^{*}}{y_{M}},$$

bearing a remarkable formal similarity with equation (3.5), which is valid for the case of uniformly distributed obstacles heights. The analogy with the uniform case extends also to the asymptotic behaviour above the threshold. Indeed, as can be appreciated from figure 4*d*, where the optimal leap angle is plotted as a function of the reduced variable $\epsilon \equiv (n-n_{c})/n_{c}$ on a log–log scale, *θ* exhibits a power law dependence on ϵ with an exponent compatible with 0.5, with a prefactor that depends on the characteristic obstacle height h*.

### 3.3. Peaked distribution of the height of the obstacles

Another simple case that deserves to be considered is when all the obstacles have the same fixed height $\tilde{h}$, namely when the height of the obstacles follows the distribution $\rho (h)=\delta (h-\tilde{h})$.

In this case, the expression of *P*_*s*,1_ is

3.9 $$P_{s,1}=\sqrt{1-\frac{1}{\sin^{2}(\theta )}\frac{\tilde{h}}{y_{M}}},$$

that leads to a *FoM* of the form

3.10 $$FoM=v_{0}\cos(\theta )\;\exp\left[n\left(\sqrt{1-\frac{1}{\sin^{2}(\theta )}\frac{\tilde{h}}{y_{M}}}-1\right)\right].$$

The maximization of this expression with respect to *θ* is quite intricate, thus we computed it numerically.

**Figure 5.**
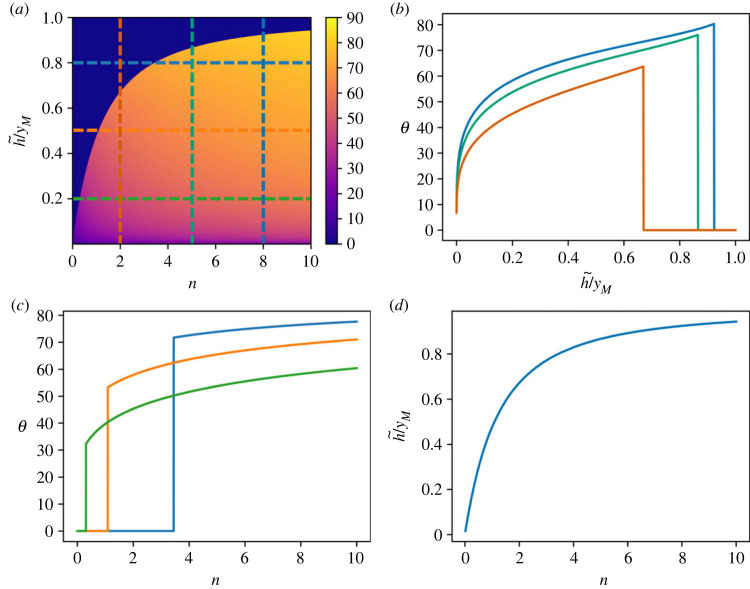
Optimal escape strategy for a peaked distribution of the obstacle height. (*a*) Optimal leap angle as a function of the average number *n* of encountered obstacles and the normalized height of the obstacles $\tilde{h}/y_{M}$. (*b*) Angle that maximizes the *FoM* plotted as a function of the normalized obstacle height $\tilde{h}/y_{M}$ for different values of the average number *n* of encountered obstacles. (*c*) Angle that maximizes the *FoM* plotted as a function of the average number *n* of encountered obstacles for different values of the normalized obstacle height $\tilde{h}/y_{M}$. (*d*) Pairs of values for the normalized obstacle height $\tilde{h}/y_{M}$ and the average number *n* of encountered obstacles where the transition occurs.

As can be appreciated from figure 5*a*, there is a region of parameters that gives *θ* = 0 as the best take-off angle, in similarity with the two precedent cases. In the region where *θ* ≠ 0, the angle *θ* increases with both $\tilde{h}/y_{M}$ (figure 5*b*) and *n* (figure 5*c*).

For each height $\tilde{h}$, there is a critical number of expected obstacles *n* under which the best strategy consists in running. The peculiar aspect of this result is that as *n* exceeds the critical value, the best angle undergoes a discrete variation and the transition is not continuous. The pairs of parameters where the transition occurs are plotted in figure 5*d*. In the presence of obstacles of height exceeding *y*_*M*_ ($\tilde{h}/y_{M}>1$), the best strategy is *θ* = 0. Under this condition the obstacles, if present, are impossible to overcome, thus the only way to maximize the *FoM* is to maximize the horizontal velocity.

A preliminary investigation of other distributions of obstacles has shown that a discontinuous transition is typical of distributions for the heights of the obstacles with a peak at a non-zero height.

### 3.4. Discussion

In both the analysed cases of uniform and exponential distributions of the heights of the obstacles, a peculiar behaviour is found: it is possible to define a critical obstacle density that depends on the length *L* of the path and on a characteristic length of the distribution of the heights of the obstacles. When the landscape is characterized by a density of obstacles lower than the critical density, the best strategy is to move horizontally without leaping, while when the density is higher than the critical density, the best take-off angle increases with the reduced density as a power law with exponent 0.5. A peaked distribution of the height of the obstacles also leads to the presence of a critical number of obstacles above which the optimal leap angle undergoes an abrupt transition to a finite non-zero value. As a general behaviour, we have found that when the average number of obstacles is fixed and the characteristic height of obstacles is increased above a typical threshold value, the optimal leap angle becomes a monotonic non-increasing function (as appreciable from figures 3*b*, 4*b* and 5*b*). This is a consequence of the fact that when the obstacles become too high the probability of overcoming them becomes small. Under these conditions, the *FoM* is maximized by smaller optimal leap angle, which allows it to achieve a faster velocity.

The variety of radically different distributions of obstacles investigated in our work across a wide area of the parameter space allows us to obtain the general and robust result that a sharp transition from running to pronking/bounding occurs irrespective of the specific features of the distribution of obstacles. However, the density of the obstacles and the distribution of their heights are input elements of our model and a more stringent test of its validity would require us to check whether the distribution of obstacles in a natural environment matches the ideal ones adopted by us. Under some circumstances, one may guess the distribution of the heights of the obstacles from prior knowledge about the environment, considering for example scale invariance, cut-offs, characteristic dimensions, etc. In some cases, measuring statistical information about the landscape is however necessary. This can happen when unknown types of obstacles (for which no prior information is available) are considered or when the statistical properties of the obstacles change in time. A solution to the problem of the determination of the distribution of obstacles in a real environment can be represented by the usage of a Digital Elevation Model (DEM) of the landscape. One of the most comprehensive digital elevation models for Earth comes from the Shuttle Radar Topography Mission [33], which gives a quasi-world wide map of elevation with a spatial accuracy of approximately 30 m and a precision on the elevation of 16 m. Similarly, the ASTER Project DEM [34] has a 99% coverage of Earth's surface with spatial resolution of 30 m and elevation accuracy around 20 m. A much more accurate DEM comes from ALOS-PRISM projects of the Japanese Space Agency (JAXA) [35], which allowed us to generate a DEM with resolution of 2.5 m and an accuracy on elevation of 5 m. These DEM projects are aimed at creating a global elevation map, thus data were mostly taken from satellites or spacecrafts. In the case of small obstacles, like grass or sand and rocks, the data from such projects are not very useful, and a most suitable way to investigate the obstacles in the environment could be the usage of a local LiDAR technology. With LiDAR one could be able to create an accurate three-dimensional map of the environment. From such a reconstruction, it is straightforward to extrapolate the density of the obstacles and the distribution of their heights. Some problems may arise when the LiDAR resolution is too large with respect to the characteristic inter-obstacle space. Moreover, if obstacles are not perfectly vertical, LiDAR inspection may fail because higher obstacles could hide smaller ones affecting the reconstruction of the heights distribution. A canopy of vegetation may easily drive to this issue. Nevertheless, if one is confident that all the obstacles have approximately the same height, the aforementioned technique can give an estimation of the unique height of the obstacles even in the critical discussed cases (like grass in [36]) and the model described in §3c can be properly applied.

Our results could provide a possible quantitative explanation of a natural phenomenon like pronking: when an animal wants to escape by moving from one place to another as quickly as possible and the environment has a density of obstacles higher than a critical density, the best survival strategy for the animal is to escape by a sequence of leaps. This model could explain the evolution of pronking/bounding in several animal species across different continents of the world. On a broader perspective, studies performed on the gait selection in black-backed jackals [37] and in domestic dogs [38] have shown that the transition between gaits is largely determined by the features of the terrain. On rugged soil frequent gait switches are required to adapt to the features of the terrain, but trot is used more frequently, thus suggesting that it represents a stable gait choice for challenging terrain, even when the substrate is covered by long grass and its features are largely unpredictable. We speculate that the adoption of a gait where at least two simultaneous footfall occur could have a common ground in determining the ability of an animal in negotiating a rugged terrain. Indeed, trot has two synchronous footfalls, where pronking and bounding have four or two, respectively. Therefore, the fact that pronking/bounding are useful to leap over obstacles could perhaps explain the shifts in gait reported in jackals and dogs, where obstacle terrain leads to shifts to gaits with simultaneous footfalls to enable better leaping and stability.

Beyond their fundamental relevance as a possible explanation for the pronking and bounding behaviour during an escape run, our results have applicative implications for robotic exploration. Robots designers look for bioinspiration in the animal world to realize robots with stable dynamic motions [17,20,24,39]. Developing control methods able to give legged robots the ability to move with agility and versatile gaits, such as real animals do, is still one of the grand challenges for robot engineers. It has been recognized that quadrupeds are the animals that run faster [21] and are able to move steadily on rough ground [24], therefore there is a special interest in reproducing their gaits. A great effort is devoted to optimizing the leg control in order to reproduce a rich variety of motions [29,32,40]. Although for our model bounding can be considered almost equivalent to pronking, this is not exactly the case for robot design. In fact among all the possible gaits, pronking is extremely promising as it allows minimizing the number of control parameters thanks to the synchronization of the extension of the four legs [18,25,26]. Conversely, the bounding gait encompasses two extra phases where only a single set of legs maintains contact with the ground [30]. Moreover, in pronking gait, pitch and roll of the animal body are minimal and therefore the model of a pronking robot does not need a control of rigid body effects, at variance with the case of a robot moving with a bounding gait [27,39]. Robots used for exploration need to be able to move in uneven terrain, a recognized specialty of quadrupeds, nevertheless only recently experiments using legged robots have started to test their performances on irregular environments [24,25,28,31,32], and to simulate their performances under reduced gravity conditions of interest for planetary exploration [41]. Most research works analysed only the motion on flat or slightly uneven terrain where the performances of wheeled rover robots are definitely superior [29]. In general, the robot gaits are evaluated on the basis of their stability, velocity and efficiency compared to the complexity of control and adaptability of the control methods. Tests of the ability of robots to overpass obstacles as a function of different gaits are very uncommon, although this approach was used for some studies on insects inspired robots [31,42]. Pierre & Bergbreiter [31] perform various experiments to test the performances of a tiny legged robot (less than 2 g in weight) in a rough artificial environment populated by randomly distributed obstacles with heights distributed normally. The robots' legs contain magnets and the motion actuation is obtained through an external magnetic field. Pronking proves to be the most effective gait to maximize the percentage of horizontal terrain explored by the robot before being stopped by an obstacle. However, when the root mean square roughness of the terrain is increased, pronking appears to be less effective than bounding, which results in it being the most effective gait. These results seem to indicate a different performance of the two gaits, not possible to predict with our model. The outcomes of this work, however, are very preliminary and would probably need to be tested in a wider range of experimental environments.

The approach proposed in our study is to connect the best leaping strategies with the statistical properties of the landscape. These relations have straightforward applications in the definition of the most suitable gait for legged robots that explore a rough terrain. When a closed-loop analysis of the landscape and the elaboration of a deterministic best strategy are excessively computationally intensive, considerations like those presented in this paper can be applied to obtain a statistically driven best open-loop strategy.

## Data Availability

The Python code used for the numerical maximization of the FoM is provided as electronic supporting material [43].
